# The Own-Race Bias for Face Recognition in a Multiracial Society

**DOI:** 10.3389/fpsyg.2020.00208

**Published:** 2020-03-06

**Authors:** Hoo Keat Wong, Ian D. Stephen, David R. T. Keeble

**Affiliations:** ^1^School of Psychology, University of Nottingham Malaysia, Semenyih, Malaysia; ^2^Department of Psychology, Macquarie University, Macquarie Park, NSW, Australia; ^3^Perception in Action Research Centre, Macquarie University, Macquarie Park, NSW, Australia

**Keywords:** other-race effect, own-race bias, multiracial, face recognition, cross-cultural

## Abstract

The own-race bias (ORB) is a reliable phenomenon across cultural and racial groups where unfamiliar faces from other races are usually remembered more poorly than own-race faces (Meissner and Brigham, 2001). By adopting a yes–no recognition paradigm, we found that ORB was pronounced across race groups (Malaysian–Malay, Malaysian–Chinese, Malaysian–Indian, and Western–Caucasian) when faces were presented with only internal features (Experiment 1), implying that growing up in a profoundly multiracial society does not necessarily eliminate ORB. Using a procedure identical to Experiment 1, we observed a significantly greater increment in recognition performance for other-race faces than for own-race faces when the external features (e.g. facial contour and hairline) were presented along with the internal features (Experiment 2)—this abolished ORB. Contrary to assumptions based on the contact hypothesis, participants’ self-reported amount of interracial contact on a social contact questionnaire did not significantly predict the magnitude of ORB. Overall, our findings suggest that the level of exposure to other-race faces accounts for only a small part of ORB. In addition, the present results also support the notion that different neural mechanisms may be involved in processing own- and other-race faces, with internal features of own-race faces being processed more effectively, whereas external features dominate representations of other-race faces.

## Introduction

The human face conveys a range of important social information, including race. Despite the proficiency with which facial information can be processed, face recognition accuracy is easily affected by race. We use the word “race” in preference to “ethnicity” throughout this article because this is the terminology used in academic, governmental, and common parlance to refer to the different community groups in Malaysia. The own-race bias (ORB; also known as the other-race effect and cross-race effect) refers to the phenomenon by which own-race faces are better recognized than faces of another race (e.g. Meissner and Brigham, 2001; Sporer, 2001; Wright et al., 2003; Walker and Hewstone, 2006a; Goldinger et al., 2009). Own-race bias has been extensively researched and is found consistently across different cultures and races, including individuals with Caucasian, African, and Asian ancestry (see Meissner and Brigham, 2001, for a meta-analytic review) and in both adults (Tanaka and Pierce, 2009; Caharel et al., 2011) and children (Anzures et al., 2014) as young as 3-month-old infants (Kelly et al., 2005; Hayden et al., 2007; Kelly et al., 2007). One early explanation for ORB was based on the hypothesis that there may be inherent physical differences in facial features between races that make discrimination easier within some races than others. However, there is no evidence to suggest on the basis of either anthropometric or behavioral data that faces from one race are physically more homogenous than faces from other races (e.g. Goldstein and Chance, 1978; Walker and Tanaka, 2003; Walker and Hewstone, 2006b).

Another common explanation for ORB appeals to the quantity of experience people have with own-race faces versus other-race faces, which is known as the contact hypothesis. According to the contact hypothesis, the amount of contact that an individual has with another race is positively correlated with the recognition accuracy for faces of that race. Wright et al. (2003) investigated ORB and interracial contact in the United Kingdom and South Africa. They employed a classic old/new recognition task in which participants were presented with 30 faces (15 African, 15 Caucasian) during the learning phase. Subsequently, they were presented with 60 faces (30 previously seen, 30 new) and were asked to indicate whether they had seen each face during the recognition phase. As predicted, ORB was observed for Caucasian participants (from the United Kingdom and South Africa), but intriguingly, African participants (from South Africa) also recognized Caucasian faces marginally better than African faces. The authors then attributed the reversal of ORB observed in African participants to the fact that their African participants were university students who had been more highly exposed to Caucasian people than most of the local South African population. Interestingly, they also discovered that African participants’ self-reported interracial contact with Caucasian people positively correlated with their recognition accuracy for Caucasian faces. Similar results were observed in a study by Fioravanti-bastos et al. (2014), which involved Caucasian and Japanese children 5 to 7 years and 9 to 11 years old born and living in Brazil. It was demonstrated that Brazilian–Japanese children did not show ORB when tested with Caucasian and Japanese faces, whereas Brazilian–Caucasian children in both age groups demonstrated ORB. Taken together, these findings suggest that the poorer recognition of other-race faces may be rooted in the amount of contact the observer has had with people of other races. The contact effect is often explained in terms of the perceptual expertise account, which proposes that more frequent interaction with own- than with other-race individuals results in richer and more differentiated cognitive representations of own-race faces [see reviews by Meissner and Brigham (2001) and Sporer (2001)], leading to greater expertise in processing and more accurate recognition of own-race faces compared to other-race faces. Although contact effects are not always evident in standard face recognition tasks, there is considerable evidence to support the hypothesis that individuating experience with own- and other-race faces contributes to ORB (Chiroro and Valentine, 1995; Kelly et al., 2007; Zhao et al., 2014). The critical role of differential experience in ORB is supported by developmental studies. Not only does ORB emerge in Caucasian and Chinese infants as young as 6 months old, but also 3-month-olds (but not newborns) even demonstrate a preference for own-race faces over other-race faces (Kelly et al., 2005, 2008; Bar-Haim et al., 2006) and discriminate between (Sangrigoli et al., 2005) own-race faces more than other-race faces. By employing a standard face recognition task, de Heering et al. (2010) found that East Asian children between 6 and 14 years of age who were adopted by European families at 2 to 26 months of age did not present a significant recognition advantage for Asian over Caucasian faces. In a similar study, Sangrigoli et al. (2005) demonstrated that Korean adults who were raised in Korea before being adopted when aged between 4 and 9 years by European Caucasian families presented a reversed ORB—they recognized Caucasian faces more accurately than Korean faces. These findings support the assumption that the face processing system is shaped by the interaction with the environment and thus can be profoundly altered by experience during early childhood.

To date, the issue regarding the role of lifetime interracial exposure in modulating ORB remains unresolved and deserves further investigation. It is worth noting that the perceptual experience hypothesis has not yet been fully explored for multiracial populations. The majority (approximately 88%) of research on ORB employed white or black populations, with only a few studies employing other races (Meissner and Brigham, 2001). In the past two decades, a growing body of research has addressed ORB in East Asian populations (e.g. Kelly et al., 2005; Goldinger et al., 2009; Wu et al., 2012). Yet, these findings may not necessarily apply to other racial groups because the social environment for individuals from a multiracial country can be more complex and vary drastically in comparison to that of individuals from a monoracial society. In this study, we focus on a key question: Does extensive exposure to faces of multiple races over a long period of time, which is not possible in a laboratory setting, augment one’s ability to recognize other-race faces?

To the best of our knowledge, studies investigating ORB in multiracial social settings are scarce (Goodman et al., 2007; Fioravanti-bastos et al., 2014; Su et al., 2017) and have produced inconsistent results. Goodman et al. (2007) demonstrated that South Africans who grew up and lived in a highly multiethnic (African–Caucasian) society did not evince a smaller ORB than did Norwegians from a predominantly Caucasian population. However, a few studies on individuals from multiracial societies have garnered some empirical support for the perceptual experience hypothesis, showing that ORB is reduced in multiracial populations, where other-race faces are frequently seen and individuated (e.g. Wright et al., 2003; Tan et al., 2012; Su et al., 2017).

### Malaysia as a Multiracial Country

Malaysia is a Southeast Asian country, but its racial composition is highly diverse, serving as a prime example of a multiracial society. Its population, which comprises racial groups of Malay (50.4%), Chinese (23.7%), Indian (7.1%), indigenous Bumiputra groups (11%), and others (7.8%; including Africans and Western–Caucasians) (Department of Statistics Malaysia, 2018), is far more racially diverse than the famously homogeneous societies of Japan and South Korea, or even those of Taiwan (with its split between indigenous Taiwanese and mainlanders) or Singapore (which has the same major race groups as Malaysia but is >75% Chinese). The high degree of racial diversity in Malaysia is also indicated by the Ethnic Fractionalization Index, an index that measures the racial (phenotypical), linguistic, and religious cleavages in society (Table 1; Yeoh, 2001). This index is based on the probability that a randomly selected pair of individuals in a society will belong to different groups [Rae and Taylor (1970); as cited in Nagaraj et al. (2015)]. The inflow of Chinese and Indian immigrant workers into Malaysia during the British colonial era led to the emergence of a multiracial characteristic of the population, with diverse religions, culture, language, and customs. The population is also highly influenced by Western culture, having been under British rule until 1957 (Kawangit et al., 2012). Considering its unique multiracial characteristics, Malaysia provides an interesting environment for face recognition research and a rich field area for studying ORB in the context of high interracial contact among the different race groups.

**TABLE 1:** Ethnic fractionalization index (EFI) of selected countries from Yeoh (2001). Higher scores represent greater racial diversity.

**Country**	**EFI**
India	0.876
Canada	0.714
Malaysia	0.694
Singapore	0.479
United States	0.395
United Kingdom	0.325
Taiwan, Republic of China	0.274
Australia	0.096
Japan	0.079
Republic of Korea	0.002

Three recent studies have highlighted the unique cultural and racial diversity in Malaysia and how this can have a direct influence on face processing ability of own- and other-race faces in children (Su et al., 2017) and young adults (Tan et al., 2012; Estudillo et al., 2019). Su et al. (2017) reported that Malaysian–Chinese children tested with four races of faces (Chinese, Malay, African, and Caucasian) showed reduced recognition of African faces, but similar recognition accuracy for Chinese, Malay, and Caucasian faces. In another study, Tan et al. (2012) reported that Malaysian–Chinese young adults performed equally well at recognizing East Asian and Western–Caucasian faces, but less well at recognizing African faces, which are not typically encountered in Malaysia. In contrast, Estudillo et al. (2019) found that Malaysians (Chinese, Malay, Indian) recognized Chinese faces equally well compared to the normative data derived from Mainland-Chinese population (Mckone et al., 2017) but showed a clear ORB for Caucasian faces. In the latter two studies, however, only Malaysian samples were involved, and conclusions were drawn without including Western–Caucasians as a comparison group.

It is also important to note that the face stimuli used in Tan et al. (2012) study were presented with the distinctive external cues (e.g. hair, ears), and those in Su et al. (2017) were presented with hairline information. Numerous studies have shown that the hair and hairline may provide high diagnostic value for unfamiliar face recognition (Ellis et al., 1979; Kramer et al., 2017). Thus, it is entirely possible that the participants based their judgment on these external diagnostic cues to achieve a generally high recognition performance across face races. We address this question through Experiment 2, which examined the relative contributions of internal and external features to own- and other-race face recognition.

### The External Features and ORB

The majority of previous research on ORB has focused on the recognition of internal features and used standard face stimuli without hair (e.g. McKone et al., 2007), likely because the internal features (i.e. eyes, nose, and mouth) have been shown to be the most significant features for face recognition, whereas external features (e.g. hairstyle and facial hair) are features that can be easily changed and therefore are potentially unreliable cues to identity. Previous studies found that the recognition and matching of unfamiliar faces rely heavily on external features (Ellis et al., 1979; Young et al., 1985; O’Donnell and Bruce, 2001), whereas familiar faces can be easily recognized and discriminated one from another based on internal features (Ellis et al., 1979; Henderson et al., 2005; Sporer and Horry, 2011; but see Toseeb et al., 2014; Toseeb et al., 2012). Some authors have argued that perceptual expertise is required to successfully encode internal face features (Megreya and Bindemann, 2009; Megreya et al., 2012; Wang et al., 2015), with developmental studies finding that adult-like processing of internal features is not achieved until between 10 and 15 years of age (Campbell et al., 1999; Want et al., 2003; but see Bonner et al., 2004).

Although there has been extensive research on the contributions of external features to face memory (Ellis et al., 1979; Jarudi and Sinha, 2003; Toseeb et al., 2012), relatively little is known about to what extent the presence/absence of external features affects ORB. This is an important consideration because the exclusion of external features may produce findings that are inconsistent with other studies using face images with external features, rendering interpretation of any differences found difficult. To our knowledge, only one study has directly tested its effect on own- and other-race recognition accuracy. Sporer and Horry (2011) tested German and Turkish participants’ recognition performance for faces from four ethnic groups: African–American, Caucasian–American, Caucasian–German, and Turkish, with the presence or absence of external features being manipulated. In the classic yes–no recognition task, both groups of participants were least accurate at recognizing African–American faces, the race group to which they would have had least exposure. The authors also reported that removing external features at encoding reduced recognition accuracy for other-race faces but not for own-race faces. However, further inspection of their data revealed that none of the significant interaction terms actually involved participant ethnicity, and the advantage for encoding the whole face over just the internal features occurred only in African–American and Turkish faces. Hence, it remains unclear how this effect may vary depending on participants’ perceptual experience with own-race versus other-race faces. In this article, we describe two experiments addressing two related questions: (1) Do participants from a multiracial country show a similar magnitude of ORB to participants from a more homogenous country? This is a test of the exposure hypothesis and is addressed in both experiments. (2) Does the inclusion/exclusion of external facial features from the stimuli influence the magnitude of ORB? This is a test of the hypothesis that participants depend more on external features for other-race than for own-race facial recognition and is addressed by comparing the results of Experiments 1 and 2.

## Experiment 1

The first experiment tested for the presence of ORB among the three main Malaysian race groups, who grew up in a highly multiracial society: Malaysian–Chinese, Malaysian–Malay, Malaysian–Indian, and a Western–Caucasian comparison group. We examined the effects of interracial contact, based on theories of ORB that attribute it to a lack of perceptual experience with other-race people. To examine whether increased other-race contact reduces cross-race differences in recognition performance, a social contact questionnaire was also used in this study to measure participants’ quantity and quality of contact with other-race people. Because of high levels of exposure to several different racial groups within their social environment, Malaysians are likely to possess discrimination abilities for multiple racial categories, especially for the faces from their own nation (e.g. Malay, Chinese, and Indian faces). We reasoned that Malaysian participants (Chinese, Malay, Indian) with sufficient multiracial experience would develop a broadly tuned face representation, such that they may show comparable recognition for all races of faces and therefore not display the traditional ORB. On the other hand, Western–Caucasian participants from a less racially diverse population are likely to show ORB—with superior performance on own-race Caucasian faces and poorer performance on other-race faces (Chinese, Malay, and Indian) with which they have less experience.

### Methods

#### Participants

Sample size was determined in advance based on previous studies that obtained a strong ORB in Malaysian–Chinese children (Su et al., 2017) and young adults (Tan et al., 2012), by using the same old/new recognition paradigm. An *a priori* power analysis was performed to determine the sample size needed to find a medium-effect size with α = 0.05 and power of β = 0.8. A total of 23 participants per group were needed to detect a medium-effect size, and a total of 16 participants per group were needed to detect a large effect size. A majority of prior studies included fewer than 25 participants per group. In Experiment 1, participants consisted of 94 young adults: 26 Malaysian–Chinese [10 males; mean age = 20.23 years (SD = 2.14)], 23 Malaysian–Malays [10 males; mean age = 19.70 (SD = 1.15) years], 22 Malaysian–Indians [11 males; mean age = 22.50 (SD = 4.35), years], and 23 Caucasians [13 males; mean age = 22.26 (SD = 3.93) years]. All Malaysian participants were university students who had not lived outside of Malaysia for more than 2 years [mean = 3.56 (SD = 5.63) months]. Caucasian participants were British exchange students who had not resided in Malaysia for more than 11/2 years [mean = 3.95 (SD = 3.42) months]. All participants had normal or corrected-to-normal vision. They gave informed consent to participate in the study, which was approved by the ethics committee of the School of Psychology at the University of Nottingham Malaysia. Each of them received either course credit or were paid RM 5 (approximately the price of a simple lunch on campus) for their participation in the study.

#### Design

The yes–no recognition task followed a 4 (face race: Chinese, Malay, Indian, and Caucasian; within-subjects) × 4 (participant race: Chinese, Malay, Indian, and Caucasian; between-subjects) mixed design. The dependent variable was the recognition sensitivity *d*′.

### Apparatus and Materials

#### Face Stimuli

**FIGURE 1:**
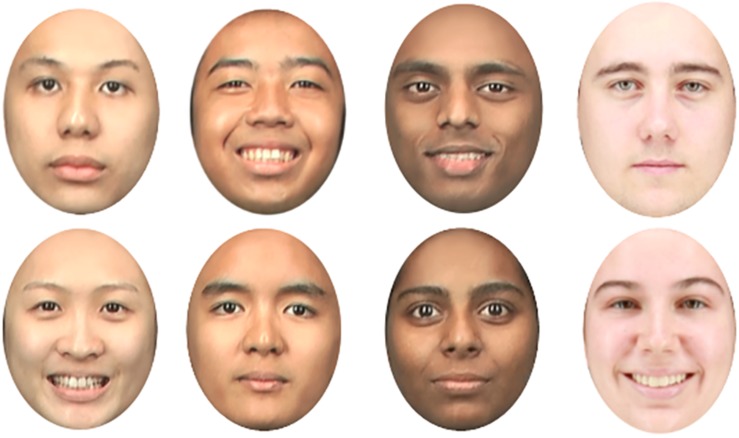
Examples of cropped stimuli with happy and neutral expressions used in Experiment 1. Each column shows a male (top) and a female (bottom) face for each race group (left to right, Malaysian–Chinese, Malaysian–Malay, Malaysian–Indian, and Western–Caucasian). The individuals depicted in this figure gave written informed consent to the publication of their images.

Photographs of 18 Chinese, 18 Malay, 18 Indian, and 18 Caucasian (half were male, half were female; different individuals to the participants completing the perceptual task) participants were taken under controlled lighting conditions, with all camera settings held constant. For each individual, two photographs were taken: one smiling and one with a neutral expression. Participants gave informed consent in writing for their images to be used in studies conducted by the researchers. Stimuli were randomly chosen to create three sets of 48 faces (12 for each face race). The original facial images were resized to 370 × 470 pixels (16-bit color depth), corresponding to a visual angle of 8.75° horizontally and 11.22° vertically at a viewing distance of 63 cm. To eliminate any confounding variations between different types of stimuli, Gaussian (radius = 3 pixels) and pixelate filters (cell size = 2 squares) in Adobe Photoshop CS6 were applied to the Caucasian facial images as an attempt to normalize the image resolution/quality. All face images were also aligned on the eyes’ position and cropped around the face in a standard oval to exclude salient cues such as ears and hairstyle. Figure 1 shows some examples of face stimuli used in Experiment 1. The stimuli were presented on a 17-inch thin-film transistor monitor with a screen resolution of 1280 × 1024 pixels. Tobii Studio experimental software was used to control the stimulus presentation.

#### Social Contact Questionnaire

The questionnaire used in this study contained 15 statements (answered once for each race group), which sought to assess quality (the first 10 items) and quantity (the last five items) of contact with individuals from the three main racial groups in Malaysia (i.e. Malay, Chinese, and Indian) and with Caucasian people. The social contact questionnaire used in this study was identical to the one used by Toseeb et al. (2012), which was a modified version of the one employed by Walker and Hewstone (2007). The questionnaire had the same items for own race and each of the other races (Appendix A). Participants rated each statement using a five-point scale, with 1 meaning “very strongly disagree” and 5 meaning “very strongly agree.”

### Procedure

Participants were first presented with eight practice trials that were identical to the rest of the experiment except that fewer faces (two for each face race; four targets and four distractors) were used to familiarize them with the task. The facial images shown in the practice phase were not used in the main experiment. The main experiment followed immediately after the practice and involved two parts: the learning and the recognition phase. In the learning phase, participants viewed 32 faces (eight Malaysian–Malay, eight Malaysian–Chinese, eight Malaysian–Indian, and eight Western–Caucasian; four males and four females for each race), one at a time. Each face was presented randomly in one of the four quadrants of the screen against a white background for 5 s, preceded by a central fixation cross with an interstimulus interval of 1 s. Participants were asked to remember as many of the faces as possible. To prevent them from using simple image matching strategies, half of the study set of each race showed a smiling expression, and the other half showed a neutral expression. For target faces, if the neutral expression was presented in the learning phase, the smiling expression was then presented in the recognition phase and vice versa.

Upon completion of the learning task, participants were given a 3-min distracter task in which they were required to complete the social contact questionnaire. In the recognition phase, participants were presented with 32 faces (including the 16 targets seen in the learning phase and 16 distractors not seen before) one at a time for 5 s each. For target faces, the facial expression changed between the study and test phases (i.e. randomized into learning and test phases) to avoid a trivial image matching strategy. The target and distractor faces were counterbalanced across participants. After viewing each face, participants were asked if they had seen the face before and chose one of the three following options: (1) yes, (2) no, or (3) yes, I definitely know this person in real life. Participants selected “yes” if they thought the face was learned (i.e. old) and “no” if they thought the face had not been presented in the learning phase (i.e. new). Furthermore, they had the option to choose an additional answer, “Yes, I definitely know this person in real life,” if they were familiar with any of the faces outside the experimental setting; for example, the individual in the stimulus was a friend of theirs or they were course mates. None of them reported knowing more than three faces from the stimulus set in real life. Individual trials with the third answer were excluded (<10% of all trials) from statistical analyses. There was no time limit for making responses via mouse clicks.

### Data Analysis

#### Recognition Sensitivity

Data from the recognition phase were sorted into four conditions for Malaysian–Chinese, Malaysian–Malay, Malaysian–Indian, and Caucasian faces: hits (correctly identified learned faces), misses (learned faces wrongly classified as new), correct rejections (new faces correctly identified as new), and false alarms (new faces wrongly classified as learned). To obtain an unbiased (e.g. strategy free) measure of people’s face recognition performance, a signal detection measure of sensitivity (*d*′) was used as the index of recognition performance (i.e. discrimination ability). To overcome infinite values of *d*′ in the case where hit rate or false alarm rate is equal to 1.0, the Snodgrass and Corwin (1988) correction factor was applied by using the following formulas:

$$\mathrm{Hit}\,\mathrm{rate}=\,\frac{\mathrm{number}\,\mathrm{of}\,\mathrm{hits}+0.5}{\mathrm{total}\,\mathrm{number}\,\mathrm{of}\,\mathrm{trials}\,\mathrm{with}\,\mathrm{signal}\,\mathrm{present}+1}$$

$$\mathrm{False}\,\mathrm{alarm}\,\mathrm{rate}=\,\frac{\mathrm{number}\,\mathrm{of}\,\mathrm{false}\,\mathrm{alarms}+0.5}{\mathrm{total}\,\mathrm{number}\,\mathrm{of}\,\mathrm{trials}\,\mathrm{with}\,\mathrm{signal}\,\mathrm{absent}+1}$$

Participants’ corrected hit and false alarm rates from the face recognition task were combined into *d*′ scores, where *d*′ is equal to *z* score for hit rates (*Z*_H_) minus *z* score for false-alarm rates (*Z*_FA_) (Macmillan and Creelman, 1991). A higher *d*′ score represents more sensitivity to a signal, whereas a score that approaches 0 represents less sensitivity.

### Results

#### Recognition Sensitivity

**FIGURE 2:**
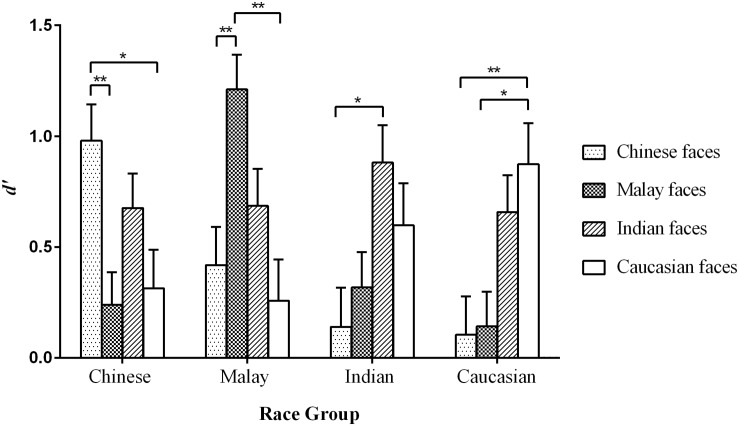
Recognition accuracy of Chinese, Malay, Indian, and Caucasian participants for own- and other-race faces in the face recognition task. Error bars represent standard errors of the mean (**p < 0.01, *p < 0.05).

To obtain an unbiased (e.g. strategy free) measure of people’s face recognition performance, recognition sensitivity *d*′ was examined. A 4 (race of observer) × 4 (race of face) mixed factorial analysis of variance (ANOVA) on *d*′ showed no significant main effect of race of observer, *F*_3_,_90_ = 1.18, *p* = 0.32, η*_*p*_*^2^ = 0.04. There was a highly significant interaction between race of observer and race of face, *F*_9_,_270_ = 5.45, *p* < 0.001, η*_*p*_*^2^ = 0.15 (Figure 2). Simple main effect analyses for the significant interaction revealed better recognition performance to own-race faces (as shown by higher *d*′ values) than to other-race faces (except for Indian faces) in Chinese, Malay, and Caucasian participants, whereas ORB was not prominent in Indian participants. In Chinese participants, there was a sensitivity advantage for recognizing own-race faces over Malay (*p* = 0.005) and Caucasian (*p* = 0.03) faces. Malay participants showed a significant ORB for Malay versus both Chinese and Caucasian faces (*p* = 0.005 and *p* = 0.002). Indian participants were significantly more sensitive to own-race faces only when compared to Chinese faces (*p* = 0.04), whereas other comparisons were non-significant but in the predicted directions. Caucasian participants were more sensitive to own-race faces than to Chinese (*p* = 0.01) and Malay faces (*p* = 0.03). In general, an own-race recognition advantage was detected for many, but not all, pairs of races.

A paired-sample *t* test showed no significant difference in the recognition accuracy of participants, *t*_91_ = 0.53, standard error of the mean = 0.09, *p* = 0.60, if they were shown the smiling or neutral expression of each face in the learning versus recognition phase.

#### Social Contact Questionnaire Responses

Amount and quality of contact were assessed using an identical version of a social contact questionnaire used by Toseeb et al. (2012). Mean scores were calculated by taking the average for each participant on both measures of quantity and quality of contact with own- and other-race groups (Table 2). Internal consistency (Cronbach’s α) was examined separately for each measure, showing a high internal reliability (α = 0.85, quantity of contact; α = 0.96, quality of contact). Two mixed factorial ANOVAs (DV = quality or quantity of contact) revealed strong interactions between race of participant, *F*_2__.__75_,_247__.__51_ = 27.31, *p* < 0.001, and race of face, *F*_3_,_270_ = 22.04, *p* < 0.001. Pairwise comparisons (Bonferroni corrected) showed that all races of participants had significantly greater amount and quality of contact with own-race than other-race people (all *p* < 0.01), except for Indian participants, who reported similar quality (but not quantity) of contact with Chinese and Malay people as with Indian people (all *p* > 0.34), implying that Indians (minority group) generally had more opportunity for other-race contact with Malay and Chinese people (majority groups).

**TABLE 2:** Means and standard errors (in parentheses) of the level of social contact (1 = no or low degree of interaction; 5 = high degree of interaction) with own- and other-race individuals reported by Chinese, Malay, Indian, and Caucasian participants.

	**Chinese participants**			**Malay participants**			**Indian participants**			**Caucasian participants**		
	**Quality of contact**	**Quantity of contact**	**Total contact**	**Quality of contact**	**Quantity of contact**	**Total contact**	**Quality of contact**	**Quantity of contact**	**Total contact**	**Quality of contact**	**Quantity of contact**	**Total contact**
Chinese people	4.55 (0.09)	4.62 (0.07)	4.60 (0.07)	3.24 (0.17)	3.10 (0.14)	3.15 (0.14)	3.28 (0.21)	3.34 (0.15)	3.32 (0.16)	2.27 (0.24)	2.34 (0.16)	2.32 (0.18)
Malay people	2.48 (0.20)	2.50 (0.16)	2.49 (0.17)	4.43 (0.15)	4.60 (0.08)	4.54 (0.10)	2.96 (0.26)	2.84 (0.20)	2.88 (0.21)	1.94 (0.20)	2.03 (0.15)	2.00 (0.16)
Indian people	2.50 (0.20)	2.34 (0.15)	2.39 (0.16)	2.70 (0.23)	2.42 (0.15)	2.51 (0.17)	4.02 (0.13)	3.98 (0.12)	3.99 (0.11)	2.26 (0.21)	2.43 (0.19)	2.37 (0.19)
Caucasian people	1.82 (0.16)	1.86 (0.11)	1.85 (0.12)	1.75 (0.17)	2.16 (0.10)	2.02 (0.11)	1.94 (0.25)	1.96 (0.16)	1.96 (0.18)	4.17 (0.17)	4.35 (0.17)	4.29 (0.15)

To reduce the possible issues of subjectivity and response bias in the self-reported data, we computed the relative scores for both quantity and quality of contact with other-race people for each race group using the following calculations:

Relative⁢quality⁢of⁢contact⁢with⁢people⁢of⁢target⁢race=

quality⁢of⁢contact⁢with⁢people⁢of⁢target⁢race-

quality⁢of⁢contact⁢with⁢own-raracce⁢people

Relative⁢quantity⁢of⁢contact⁢with⁢people⁢of⁢target⁢race=

quantity⁢of⁢contact⁢with⁢people⁢of⁢target⁢race-

quantity⁢of⁢contact⁢with⁢own-race⁢people

In addition, to control for individual differences in face recognition ability, we calculated the size of ORB in recognition memory for each participant by subtracting *d*′ scores for target-race faces from *d*′ scores for own-race faces. Pearson correlations were calculated between own-race recognition advantage and relative scores. Based on the contact hypotheses, we anticipated that individuals who reported higher levels of interracial contact would show a smaller ORB in comparison to those who reported less interracial contact. Negative correlations between these two measures were expected. However, Pearson correlation analyses revealed that all correlations failed to surpass the Bonferroni-corrected α of 0.002 (0.05/24), two-tailed, and a few were even in the opposite-to-predicted direction (Table 3). This suggests that both self-reported quantity and quality of contact with other-race individuals did not consistently predict how well other-race faces would be recognized.

**TABLE 3:** Correlations between relative contact scores and own-race recognition advantage (d′ scores for own-race faces - d′ scores for other-race faces) for Chinese, Malay, Indian, and Caucasian participants.

**Race of participants**	**Relative quantity of contact with Chinese**	**Relative quality of contact with Chinese**	**Relative quantity of contact with Malays**	**Relative quality of contact with Malays**	**Relative quantity of contact with Indians**	**Relative quality of contact with Indians**	**Relative quantity of contact with Caucasians**	**Relative quality of contact with Caucasians**
Chinese	–	–	*r* = 0.45, *p* = 0.02	*r* = 0.04, *p* = 0.84	*r* = 0.16, *p* = 0.45	*r* = −0.06, *p* = 0.78	*r* = −0.07, *p* = 0.72	*r* = −0.13, *p* = 0.54
Malay	*r* = −0.18, *p* = 0.41	*r* = 0.09, *p* = 0.69	–	–	*r* = −0.24, *p* = 0.26	*r* = −0.47, *p* = 0.02	*r* = −0.17, *p* = 0.45	*r* = −0.21, *p* = 0.34
Indian	*r* = 0.30, *p* = 0.18	*r* = 0.40, *p* = 0.07	*r* = 0.57, *p* = 0.006	*r* = −0.07, *p* = 0.75	–	–	*r* = −0.20, *p* = 0.37	*r* = −0.08, *p* = 0.72
Caucasian	*r* = −0.18, *p* = 0.41	*r* = −0.03, *p* = 0.90	*r* = −0.28, *p* = 0.20	*r* = −0.24, *p* = 0.26	*r* = 0.10, *p* = 0.64	*r* = 0.06, *p* = 0.80	–	–

*Shaded cells indicate significant correlation *r* values (at α = 0.05) before Bonferroni corrections.*

### Discussion

The current study is the first to investigate ORB among four different race groups: Malaysian–Chinese, Malaysian–Malay, Malaysian–Indian, and Western–Caucasian, who had differential exposure to other races in a racially diverse country. One explanation offered for ORB suggests that processing of facial information can be improved through contact with others. We reasoned that if this generalized version of the contact hypothesis were true, Malaysian groups from a multiracial population would be able to develop a facial representation that was broadly tuned to optimally encode individuating facial information from different races, such that they might exhibit a reduced ORB relative to the Western–Caucasian participants. However, our results do not support this hypothesis.

Own-race bias was found in all race groups: when presented with face images containing only internal features, Malaysian participants (Chinese Malay, Indian) showed a recognition deficit for other-race faces. Chinese participants recognized Malay and Caucasian faces significantly more poorly than own-race faces. Broadly similar to Chinese participants, Malay participants exhibited an ORB in favor of own-race faces, showing higher ability to recognize own-race faces compared to Caucasian and Chinese faces. Caucasian participants were found to be better at recognizing own-race faces than Chinese and Malay faces. Own-race bias was less pronounced in Indian participants compared to other groups, with significant recognition deficit only for Chinese faces, and non-significant differences in the predicted directions for other-race faces.

It should be noted that regardless of race group participants recognized Indian faces fairly well, possibly due to greater distinctiveness of the Indian face set. To test this possibility, we conducted a follow-up study where the facial distinctiveness of the stimulus sets was measured based on the mean subjective ratings obtained not only from same-race raters, but also from other-race raters (Supplementary Table 1). The results revealed that the Indian faces selected in the present study were more distinctive from one another than the other three races of faces. This might have hindered a shift toward significantly lower level of recognition performance for Indian faces than for own-race faces in Chinese, Malay, and Caucasian participants. Thus, any result derived from the Indian faces should be interpreted with caution.

Our findings differ from the previous two studies conducted in a Malaysian population (Tan et al., 2012; Su et al., 2017). Su et al. (2017) found that Malaysian–Chinese children tested with four races of faces (Chinese, Malay, African, and Caucasian) showed reduced recognition of African faces, but similar recognition accuracy for Chinese, Malay, and Caucasian faces. This study differed from the current study in a number of ways: first, it used children (5- and 6-year-olds and 13- and 14-year- olds) rather than adults; second, the number of faces to remember and the number of distractor faces were smaller; third, the stimuli included parts of the external facial features (the outline of the face and hairline). The relative easiness of the task renders these findings not directly comparable with our young adult samples. In another study, Tan et al. (2012) found that Malaysian–Chinese young adults performed equally well at recognizing East Asian and Western–Caucasian faces. However, the stimuli included the external features (e.g. hair, facial contour) of the face, which have been shown to be relied on more during other-race face recognition (Sporer and Horry, 2011). Experiment 2 was therefore conducted to examine the role of external features in ORB.

## Experiment 2

In Experiment 1, participants showed an ORB when only information about the internal features was available. The exclusion of external features from the cropped facial stimuli could have somehow increased the difficulty of the recognition task (Ellis et al., 1979; Jarudi and Sinha, 2003; but see Toseeb et al., 2012), strengthening ORB effects. In Experiment 2, we further examined whether and, if so, to what extent the presence of external features reduces ORB in face memory, including both Malaysian and Caucasian samples. In this experiment, we replicated Experiment 1 using the identical procedure but different stimulus type. Rather than faces cropped to the internal features using egg-shaped masks, face images including the external features were presented. Given that external features are useful for recognizing and matching unfamiliar faces (Ellis et al., 1979; Endo et al., 1984; Young et al., 1985; O’Donnell and Bruce, 2001), we predicted that adding the external features would improve recognition accuracy. Previous research has shown that participants are less efficient at processing configural relationships between internal facial features of other-race faces than own-race faces (e.g. Rhodes et al., 2009). This could lead to a reliance on processing of external facial features of other-race faces. Hence, we hypothesized that the presence of external features would substantially enhance participants’ performance for other-race faces more than for own-race faces, reducing ORB compared to Experiment 1.

### Methods

#### Participants

A separate group of undergraduate students participated in this experiment. There were 23 Malaysian–Chinese [13 males; mean age = 21.52 (SD = 3.76) years], 23 Malaysian–Malays [10 males; mean age = 19.74 (SD = 3.76) years], 25 Malaysian–Indians [12 males; mean age = 20.92 (SD = 5.60) years], and 20 Western–Caucasians [10 males; mean age = 23.05 (SD = 4.39) years]. All had normal or corrected-to-normal vision. All participants gave informed consent to participate in the study. *A priori* power analysis showed that, for all of the within-between interaction terms that directly related to our hypotheses, this sample size gave sufficient power to detect medium-effect sizes of η*_*p*_*^2^ < 0.06, with α = 0.05, and power (1 - β) = 0.80.

#### Materials

**FIGURE 3:**
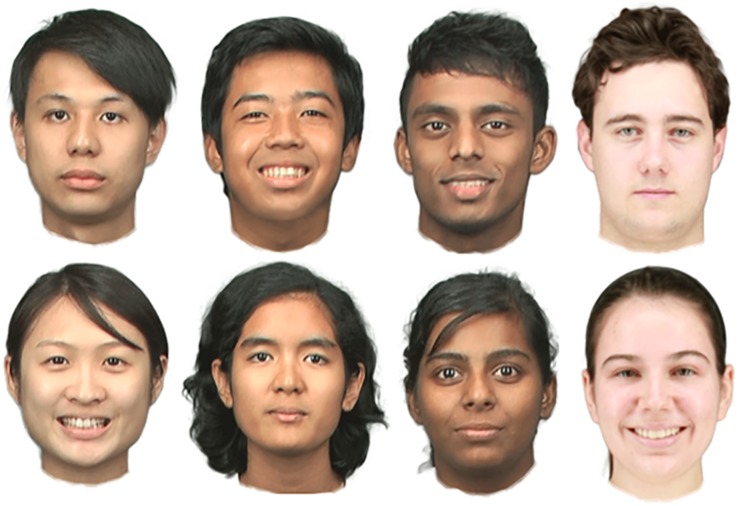
Examples of whole-face stimuli with smiling and neutral expressions used in Experiment 2. Each column shows a male and a female face for each race group (Malaysian–Chinese, Malaysian–Malay, Malaysian–Indian, and Western–Caucasian, respectively). The individuals depicted in this figure gave written informed consent to the publication of their images.

We employed the same set of stimuli as in Experiment 1, but presented with the external features and hair being retained. Other aspects of the stimuli were identical to Experiment 1. Examples of stimuli are shown in Figure 3.

#### Procedure

Experiment 2 followed the same procedure as Experiment 1. In both experiments, participants performed a yes–no recognition task for four face races: Malaysian–Chinese, Malaysian–Malay, Malaysian–Indian, and Western–Caucasian.

### Results

#### Recognition Sensitivity

A 4 (race of face: Malay, Chinese, Indian, or Caucasian) × 4 (race of observer: Malay, Chinese, Indian, or Caucasian) mixed factorial ANOVA revealed that *d*′ scores did not differ significantly between races of faces, *F*_2__.__55_,_222__.__06_ = 0.87, *p* = 0.46, η*_*p*_*^2^ = 0.01, Greenhouse–Geisser corrected, as well as between races of observers, *F*_3_,_87_ = 1.95, *p* = 0.13, η*_*p*_*^2^ = 0.05. The interaction between race of face and race of observer also did not reach significance, *F*_9_,_261_ = 1.57, *p* = 0.12, η*_*p*_*^2^ = 0.06, indicating the absence of ORB among different race groups.

#### Does Performance Improve in the Whole Face Condition?

In Experiment 2, participants performed equally well at recognizing own- and other-race faces, suggesting that their recognition performance was influenced by the inclusion of external features. Next, we conducted additional analyses on the *d*′ scores to explore whether the magnitude of ORB (i.e. the recognition performance for own- and other-race faces) significantly differed between the egg-shaped (Experiment 1) and full-face (Experiment 2) conditions. A 2 (face type: egg-shaped vs. whole) × 4 (race of observer: Chinese, Malay, Indian, and Caucasian) × 4 (face race: Chinese, Malay, Indian, and Caucasian) mixed factorial ANOVA was used to identify any significant main effects and interactions related to face type—where race of observer and face type were between-subject factors, and face race was within-subjects factor. A highly significant main effect of face type, *F*_1_,_177_ = 61.21, *p* < 0.001, η*_*p*_*^2^ = 0.26, was accompanied by a marginally significant interaction between face type and race of observer, *F*_3_,_177_ = 2.32, *p* = 0.08, η*_*p*_*^2^ = 0.08. Simple main effect analyses revealed that most race groups (Chinese, Indian, and Caucasian) performed significantly better in the whole-face than egg-shaped condition (all *p* < 0.001), and there was a marginally significant difference in the Malay group (*p* = 0.07).

**FIGURE 4:**
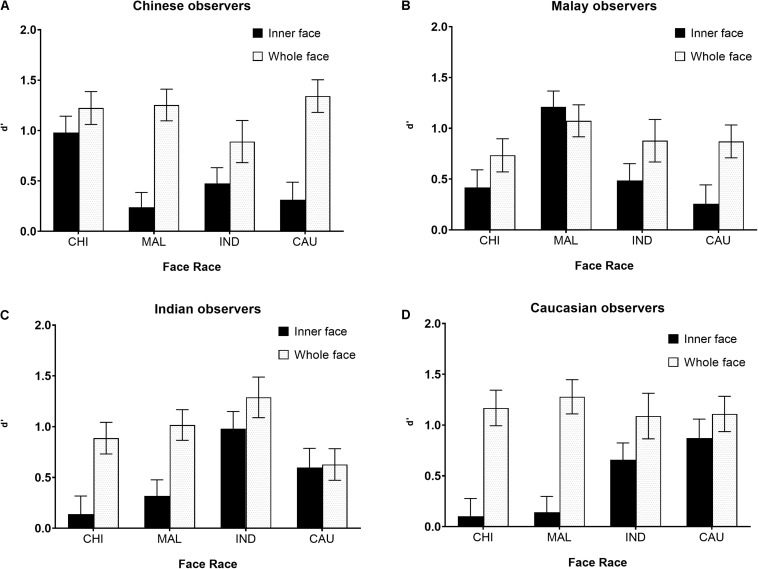
*d*′ for Chinese, Malay, Indian, and Caucasian faces in Chinese **(A)**, Malay **(B)**, Indian **(C)**, and Caucasian **(D)** participants in the recognition task. Face recognition was impaired for face images without external features. Surprisingly, however, the absence of external features led to a greater decline in the participants’ ability to recognize other-race faces. Error bars represent standard error of the mean.

Interestingly, there was a significant three-way interaction involving face type, face race, and race of observer (see Figure 4 for means and standard errors), *F*_9_,_531_ = 3.21, *p* = 0.001, η*_*p*_*^2^ = 0.05. Separate analyses for the four observer races revealed that the interaction between face type and face race was significant for all the race groups. In Chinese participants, the presence of external features substantially improved their recognition of Malay and Caucasian faces (both *p* < 0.001) but not for Chinese (*p* = 0.26) and Indian faces (*p* = 0.40). Malay participants performed better in whole-face trials compared to egg-shaped trials only for Caucasian faces (*p* = 0.03) but not Chinese (*p* = 0.14), Malay (*p* = 0.50), and Indian faces (*p* = 0.51). Indian participants recognized Chinese (*p* = 0.005) and Malay faces (*p* = 0.005) significantly better in the whole-face condition, whereas no difference was found for Indian and Caucasian faces (both *p* > 0.05). Caucasian participants showed higher accuracy in the whole-face condition for Chinese and Malay faces (both *p* < 0.001), and a marginally significant effect for Indian faces (*p* = 0.07), but no difference for Caucasian faces (*p* = 0.32). Upon close examination, there was a general trend for the presence of external features to affect own-race face recognition to a smaller extent as compared to other-race faces in all race groups. We discounted the possibility that the lack of improvement for own-race face recognition was merely due to ceiling effects as the *d*′ for own-race faces were not significantly negatively skewed (Supplementary Table 2).

#### Social Contact Questionnaire Responses

As in Experiment 1, the social contact questionnaire administered to these race groups showed that their performance did not positively correlate with self-reports of the quality and quantity of contact they had with own- versus other-race individuals.

### Discussion

In Experiment 2, we further investigated the effect of external features on the magnitude of ORB by employing whole-face stimuli with external features. It was predicted that adding the external features would improve recognition accuracy, as external features are useful for recognizing unfamiliar faces (Axelrod and Yovel, 2010; Sporer and Horry, 2011). Consistent with our hypothesis, the results showed that ORB disappeared when participants were presented with whole faces with external features but not when presented with only internal features, suggesting that the external features provide important input information. Given the significant role of internal features in the representation of familiar faces compared to unfamiliar faces (Ellis et al., 1979; Megreya and Bindemann, 2009), we also hypothesized that such internal feature advantage could extend to faces of familiar race versus unfamiliar race. This hypothesis received strong support in a highly significant three-way interaction involving face type, face race, and race of observer. Our results showed that the presence of external features (compared to internal features only) did not significantly improve recognition of own-race faces, but did increase recognition accuracy for other-race faces, suggesting a greater dependence on external features for other-race processing and on internal features for own-race face processing.

Several face recognition studies employing face images with external features have reported evidence of ORB (Wright et al., 2003; Tanaka and Pierce, 2009; Tan et al., 2012; Su et al., 2017). For instance, ORB was found for African faces in Malaysian–Chinese children (Su et al., 2017) and young adults (Tan et al., 2012). Yet, our Experiment 2 failed to uncover this effect. The discrepant findings could stem from methodological differences between studies. Tan et al. (2012) and Wright et al. (2003) used identical pictures in the study and test phases, whereas Tanaka and Pierce (2009) placed internal features in a standard face template with identical hairstyle and facial contour. Our recognition task differed from these studies, in that we used different photographs (with changes in expression) of the same individuals at study and test phases in order to promote recognition strategies based on internal facial features.

Previous research has shown that participants are less accurate at processing internal features (Rhodes et al., 2009; Hayward et al., 2017) and configural relationships between internal features (Tanaka et al., 2004; Michel et al., 2006) of other-race faces than own-race faces. In the current experiment, expression changes between the study and test phases might have increased the task difficulty to encode configural information from internal features. Furthermore, given that our whole-face sets include external features with a considerable degree of within-race variability, this might have increased participants’ tendency to adopt non-face strategies based on external cues and consequently masked ORB in face memory.

Our results bring an interesting perspective to our understanding of the mechanisms by which own- and other-race faces are processed; own-race face recognition is less affected by the absence/presence of external features, likely due to the precision and flexibility of facial representations. A greater reliance on the processing of external features of other-race faces was demonstrated through a clear reduction in recognition accuracy when external features were removed. Here we suggest that recognition differences between own-race and other-race faces may be related to the efficacy of feature encoding, with internal features of own-race faces being processed more efficiently, whereas external features dominate representations of other-race faces.

## General Discussion

While the vast majority of previously published ORB studies have been conducted in less racially diverse communities (e.g. United States, United Kingdom, South Africa, and Germany) than Malaysia, recognition deficits for other-race faces have often been attributed to the amount of contact with other-race and own-race faces. The present study examined whether ORB is also present in a highly multiracial society, namely, Malaysia, in which individuals generally have increased day-to-day direct exposure to other-race faces. Several studies have shown that sufficient contact with other-race people can ameliorate ORB (Wright et al., 2003; Walker and Hewstone, 2006b; Fioravanti-bastos et al., 2014); yet, some studies have demonstrated that substantial interracial contact does not necessarily ensure that other-race face recognition will improve (e.g. Cross et al., 1971; Ng and Lindsay, 1994; Walker and Hewstone, 2006a; Jackiw et al., 2008; Rhodes et al., 2009).

In Experiment 1, ORB was manifested in terms of better recognition sensitivity for own-race faces, not only in Caucasian participants, but also in Malaysian participants (Malay, Chinese, and Indian) who grew up in a racially diverse environment. These results clearly failed to support our prediction, derived from the contact hypothesis, of equally high recognition performance for own- and other-race faces in each group of Malaysian participants. Surprisingly, it seems that even Malaysian young adults had difficulty generalizing their perceptual expertise for own-race faces to other-race faces they frequently encountered in a multiracial environment. This adds to a body of evidence indicating that ORB in adulthood is a very robust effect and may not be as malleable as commonly assumed (Tanaka et al., 2013).

Our finding also raises an open question of why ORB seems to be reduced in face training studies (Hills and Lewis, 2006; Heron-Delaney et al., 2011; Anzures et al., 2013) but not in a multiracial environment. One explanation is that more “natural” face experience may not function in the same way as these laboratory manipulations. Most laboratory training methods only increase other-race face experience quantitatively via photographic exposure in extensive, intentional face learning tasks, which differ from casual individuating experience with faces from other races in the real world. Although training studies provide an indication regarding the flexibility of ORB as well as the plasticity of face recognition systems (Hills and Lewis, 2006; DeGutis et al., 2011; Hills and Lewis, 2011; Tanaka et al., 2013), the enhancement effect from training studies is often transient (e.g. Hills and Lewis, 2011).

The relationship between other-race contact and ORB in Malaysian and Caucasian samples was further assessed by examining the pattern of correlations between self-rated interracial contact and recognition performance of other-race faces. According to the contact hypothesis, the amount of contact that an individual has with another race should be positively correlated with the accuracy of recognizing individuals from that race. In the two experiments, although demographics imply that Malaysian participants generally had a considerable amount of exposure to other-race people, the multiple correlation analyses revealed that neither relative quantity nor quality of interracial contact predicted the magnitude of ORB. In fact, meta-analysis of ORB studies revealed that self-report measures of other-race contact accounted for less than 3% of the total variance found in ORB (Meissner and Brigham, 2001). The very modest contact effects typically found imply that interracial contact may not be one of the critical factors that mitigate ORB. Rather, it may just play a small, mediating role in ORB.

Another possibility for the lack of contact effect on ORB might be due to the measurement we used. As the social contact questionnaire did not separate past experience from current experience participants had with other-race people, it remains possible that the malleability of ORB is determined by the age at which experience with another racial group begins. This argument is supported by evidence from developmental studies showing that infancy (Liu et al., 2015; Chien et al., 2016; Singarajah et al., 2017) and childhood (Sangrigoli et al., 2005; de Heering et al., 2010; Su et al., 2017; Mckone et al., 2019) are sensitive periods beyond which the effect of experience on face recognition is markedly reduced. Future studies using a different contact questionnaire that distinguishes past from current interracial experience may offer a greater potential for revealing the link between perceptual expertise across development and plasticity of other-race face recognition.

Despite many years of interracial experience, ORB in Malaysian young adults does not seem to be attenuated or eliminated as compared to Western–Caucasian young adults who lived in communities that are relatively less racially and ethnically heterogeneous. This unexpected finding renders previous reports that ORB is less evident in multiracial populations (Chiroro and Valentine, 1995; Wright et al., 2001; Bar-Haim et al., 2006), can be reversed following cross-race adoption before the age of 9 years (Sangrigoli et al., 2005), and can be reduced by training (Hills and Lewis, 2006) difficult to interpret. Nevertheless, our data lead us to consider whether, in addition to interracial contact *per se* affecting the magnitude of ORB, the ability to recognize other-race faces might also be affected by the age at which that contact is obtained. Although the current study was not specifically designed to address this question as the period of contact was not clearly measured, it raises the intriguing possibility that Malaysian individuals living in a racially heterogeneous context might still lack childhood experience in individuating other-race individuals due to prototypical perceptual environmental in the early developmental stages of face recognition ability. In fact, there is evidence that Malaysians interact with other races less when they are children than when they are adults. The low level of interracial contact during infant and childhood in Malaysians is commonly reflected through same-race primary caregivers (Su et al., 2018) and the racially segregated educational systems in primary and secondary schools (Kawangit et al., 2012). For instance, Kawangit et al. (2012) reported that the Chinese usually sent their children to Chinese schools with their syllabi adopted from Mainland China; Malays sent their children to Madrasa (religious schools), and Indians to Tamil schools. Therefore, opportunities for Malaysians (Malays, Chinese, and Indian) to integrate and interact with other-race individuals as a community in early childhood may not be as frequent as expected.

Our conjecture that ORB might be specifically associated with low childhood contact would imply that the effectiveness of multiracial experience on tuning the adult face recognition system would differ, depending on individuals’ early other-race experience. This idea parallels two concepts in existing literature: first, a sensitive period in children’s language development in which second-language learning is better if exposure occurs earlier in development rather than later (Norrman and Bylund, 2016); ORB in face recognition may stem from a mechanism analogous to the language-familiarity effect (Fleming et al., 2014); and second, evidence of an infancy-specific exposure influence on ORB for faces (i.e. perceptual narrowing in which 3-month-olds can individuate faces from multiple races, and even non-human primate species, but 9-month-olds can individuate only own-race faces; Pascalis et al., 2001; Kelly et al., 2009, 2007). Hence, changes in perceptual experience during the critical period of the development of ORB may play a crucial role in reorganizing the face representation to adapt to changes in multiracial experience in adulthood (Mckone et al., 2019).

Recent developmental studies have demonstrated a critical period for plasticity of ORB (Su et al., 2018; Mckone et al., 2019), and such recognition bias can be reduced by contact with the faces of another race during childhood (e.g. Sangrigoli et al., 2005; de Heering et al., 2010), pointing toward the importance of early individuating experience in developing mechanisms of remembering and distinguishing other-race faces. Perhaps such early formed recognition bias for own-race faces cannot be readily altered by increased exposure to other-race faces in adulthood. Future studies should further investigate the relative contributions of early and late interracial experiences to the reduction of ORB.

Another open question is whether people possess the necessary perceptual abilities to recognize other-race faces at the level of the individual, but only lack the social motivation to do so (Hugenberg et al., 2007). According to the social–cognitive models (e.g. Sporer, 2001), the source of ORB is not perceptual, but a resistance to individuate other-race faces due to their out- group status. Hence, the emergence of ORB may be due to motivational factors rather than to changes in perceptual expertise. Alternatively, ORB could be a product of converging factors involving social categorization, motivated individuation, and perceptual experience; for example, neither raw perceptual exposure nor the motivation to individuate is sufficient to attenuate ORB but requires both the proper motivation and practice to individuate other-race faces. Further research is required to confirm this hypothesis.

In addition to investigating ORB in multiracial context, the current study also bears on the issue of whether the contribution of external features changes as a function of perceived face race. In Experiment 2, we demonstrated that ORB disappeared when faces were presented with external features, suggesting that external features play a larger role in the recognition of other- than own-race faces (Sporer and Horry, 2011). Our current findings are compatible with the in-group/out-group model proposed by Sporer (2001), suggesting that perception of own- race faces automatically initiates a finer level of perceptual encoding processing that emphasizes the internal features of a face that helps distinguish the target from similar faces in memory. In contrast, other-race face perception promotes a categorization process that accentuates race-specific features at the expense of individuating information.

Experiment 2 provides strong support for the idea that external features, which comprise featural characteristics of information, play a more important role in other-race face recognition. While most face recognition studies have ignored the additional effects of external features, there is increasing evidence from behavioral and neuroimaging studies suggesting that external features are encoded alongside internal features within a holistic face representation (Jarudi and Sinha, 2003; Andrews et al., 2010; Axelrod and Yovel, 2010). As a result, although accurate face recognition can be achieved when only the internal features are presented (e.g. Anaki and Moscovitch, 2007), altering external features can sometimes disrupt face recognition (Fletcher et al., 2008; Toseeb et al., 2012). A more fruitful avenue for future research may be to investigate the extent to which altered external and/or internal features differentially affect facial representation of own- and other-race faces.

The current study has significant methodological implications for research in face recognition. The limited ecological validity of the laboratory use of stimuli displaying only internal features has been addressed and criticized frequently because the generalizability of the obtained results is questionable (e.g. Kelly et al., 2011). Results based on face images free from any cropping (e.g. with hair and facial contours included) are arguably more representative of performance in real-world viewing conditions; however, we suggest that using this type of stimulus may encourage reliance on external features for other-race faces (Megreya and Bindemann, 2009; Sporer and Horry, 2011), thereby masking the genuine ORB in face memory. In contrast, cropped face images without external features may accentuate the ability to process configural information from faces. Because the removal of external features is more detrimental to the recognition of other- than own-race faces, a face memory task that utilizes internal-features-only faces could be more sensitive in picking up a potentially subtle ORB. Because very different conclusions could have been drawn from studies due to the selection of stimulus type, future replications of ORB should take the stimulus type into account when comparing the results between studies.

In summary, this cross-racial study demonstrates that ORB in face recognition remains evident not only in Western–Caucasian participants, but also in Malaysian individuals who live in a highly multiracial population. It appears that Malaysians’ substantial everyday exposure to different races does not necessarily help in developing a broadly tuned representation that accommodates multiple other-race faces. The results converge with existing literature to suggest that there is relatively little plasticity in face recognition in adulthood (e.g. Singh et al., 2017; Tree et al., 2017). Given the existing evidence that Malaysians interact with other races less when they are children (Kawangit et al., 2012; Su et al., 2018), the robustness of ORB in the multiracial population implies a relative lack of perceptual experience with other-race faces during childhood (de Heering et al., 2010; Su et al., 2018; Mckone et al., 2019; see also Sangrigoli et al., 2005). Additionally, the magnitude of ORB was modulated by the presence/absence of external features, such that other-race faces without external features were recognized poorly. This finding not only highlighted the significant methodological implications for ORB research, but also shed further light on the face representations and mechanisms that govern own- versus other-race face recognition. People encode and/or retrieve own- and other-race faces from memory in qualitatively different ways, with internal features of own-race faces being processed more effectively, whereas external features dominate representations of other-race faces.

## Data Availability Statement

All datasets generated for this study are included in the article/Supplementary Material.

## Ethics Statement

The studies involving human participants were reviewed and approved by the Ethics committee of the School of Psychology at the University of Nottingham Malaysia. The patients/participants provided their written informed consent to participate in this study.

## Author Contributions

HW, IS, and DK contributed to the conception and design of the study. HW collected the face stimuli and performed the statistical analysis and wrote the first draft of the manuscript. HW and IS wrote sections of the manuscript. All authors contributed to the manuscript revision, read and approved the submitted version.

## Conflict of Interest

The authors declare that the research was conducted in the absence of any commercial or financial relationships that could be construed as a potential conflict of interest.

## Appendix A

Social Contact Questionnaire.

Gender: Male/Female, Race:, Age:, and Participant Number:

**Table:** For each of the statements listed below please identify to what extent you agree for each of the three groups of people.

	**Malay people**					**Chinese people**					**Indian people**					**White people**				
I often work closely (voluntary/paid work/studied) with	1	2	3	4	5	1	2	3	4	5	1	2	3	4	5	1	2	3	4	5
Within my circle of friends I regularly socialize with	1	2	3	4	5	1	2	3	4	5	1	2	3	4	5	1	2	3	4	5
My closest friends are	1	2	3	4	5	1	2	3	4	5	1	2	3	4	5	1	2	3	4	5
In my family I have many	1	2	3	4	5	1	2	3	4	5	1	2	3	4	5	1	2	3	4	5
When having problems with my work I usually ask for help from	1	2	3	4	5	1	2	3	4	5	1	2	3	4	5	1	2	3	4	5
On a regular basis I interact with	1	2	3	4	5	1	2	3	4	5	1	2	3	4	5	1	2	3	4	5
For a prolonged period of time I have lived in the same house as	1	2	3	4	5	1	2	3	4	5	1	2	3	4	5	1	2	3	4	5
I regularly spend time at the homes of	1	2	3	4	5	1	2	3	4	5	1	2	3	4	5	1	2	3	4	5
I have often comforted/have been comforted by	1	2	3	4	5	1	2	3	4	5	1	2	3	4	5	1	2	3	4	5
I frequently give/receive personal advice to/from	1	2	3	4	5	1	2	3	4	5	1	2	3	4	5	1	2	3	4	5
I know lots of	1	2	3	4	5	1	2	3	4	5	1	2	3	4	5	1	2	3	4	5
For a prolonged period of time I have lived on the same street as	1	2	3	4	5	1	2	3	4	5	1	2	3	4	5	1	2	3	4	5
In the media I regularly see	1	2	3	4	5	1	2	3	4	5	1	2	3	4	5	1	2	3	4	5
In my current occupation I come across many	1	2	3	4	5	1	2	3	4	5	1	2	3	4	5	1	2	3	4	5
Whilst socializing I come across many	1	2	3	4	5	1	2	3	4	5	1	2	3	4	5	1	2	3	4	5
