# Supplementary material for: The Own-Race Bias for Face Recognition in a Multiracial Society
Source: Front Psychol. 2020 Mar 6;11:208. doi: 10.3389/fpsyg.2020.00208 (PMC7067904; doi:10.3389/fpsyg.2020.00208)
Supplement: Supplementary file 1 [file Table_1.DOCX]

# Supplementary materials

Supplementary Table 1.

*Mean similarity ratings for Chinese, Malay, Indian, and Caucasian faces by the four race groups*

|  | Chinese (N=24) | Malay (N=24) | Indian (N=25) | Caucasian (N=19) |
| --- | --- | --- | --- | --- |
| Chinese faces | 3.09 (0.60) | 3.48 (0.66) | 3.14 (0.66) | 3.55 (0.74) |
| Malay faces | 3.30 (0.99) | 3.45 (0.86) | 3.04 (0.94) | 3.36 (0.79) |
| Indian faces | 3.45 (1.17) | 3.58 (1.18) | 3.05 (1.25) | 3.25 (1.15) |
| Caucasian faces | 4.00 (0.74) | 3.78 (0.76) | 3.08 (0.92) | 3.54 (0.95) |

*Note.* Ratings were on a scale from 1 (extremely different) to 7 (extremely similar). Standard deviations are in parentheses. Pairwise comparisons revealed that the mean similarity ratings for Indian faces (*M*=3.08) differed significantly from Chinese (*M*=3.46), Malay (*M*=3.57), and Caucasian faces (*M*=3.42) (all p <.001).

Supplementary Table 2.

*Skewness and Kurtosis of own-race d’ (with standard error of mean) for each race group of observers*

|  | Experiment 1 | |  | Experiment 2 | |
| --- | --- | --- | --- | --- | --- |
| Race of observers | M*_skewness_* | Kurtosis |  | M*_skewness_* | Kurtosis |
| Chinese | -0.25 (0.46) | -1.14 (0.89) |  | -0.66 (0.48) | -0.74 (0.94) |
| Malay | -0.17 (0.48) | -1.33 (0.94) |  | -0.44 (0.48) | 0.54 (0.94) |
| Indian | -0.32 (0.49) | -0.59 (0.95) |  | -0.06 (0.46) | -0.94 (0.90) |
| Caucasian | 0.40 (0.48) | -0.29 (0.94) |  | -0.39 (0.51) | -1.39 (0.99) |
